# Supplementary material for: Non-Markovian recovery makes complex networks more resilient against large-scale failures
Source: Nat Commun. 2020 May 19;11:2490. doi: 10.1038/s41467-020-15860-2 (PMC7237476; doi:10.1038/s41467-020-15860-2)
Supplement: Supplementary file 1 — Supplementary Information [file 41467_2020_15860_MOESM1_ESM.pdf]

Supplementary Information for

Non-Markovian recovery makes complex networks more resilient against large scale failures

Zhao-Hua Lin, Mi Feng, Ming Tang, Zonghua Liu, Chen Xu, Pak Ming Hui and Ying-Cheng Lai

Corresponding author: Ming Tang (tangminghan007@gmail.com), Zonghua Liu(zhliu@phy.ecnu.edu.cn)

CONTENTS

I. Supplementary Figures 2

II. Supplementary Notes 11
- A. Supplementary Note 1: Pairwise approximation theory 11
  - 1. Markovian recovery model 11
  - 2. Non-Markovian recovery model 14
  - 3. Relationship between MR and NMR models 17
- B. Supplementary Note 2: Effects of different initial conditions on failure propagation and evolutionary trajectories of failed nodes 18
- C. Supplementary Note 3: Markovian and non-Markovian dynamics when external recovery is slower than internal recovery 19
- D. Supplementary Note 4: Effects of network structure on Markovian and non-Markovian recovery dynamics 19
  - 1. Effects of degree-degree correlation 20
  - 2. Effects of community structure 21
- E. Supplementary Note 5: Markovian and non-Markovian recovery dynamics in empirical networks 22
- F. Supplementary Note 6: Markovian and non-Markovian recovery dynamics in power-grid synchronization 23

III. Supplementary References 24

I. SUPPLEMENTARY FIGURES

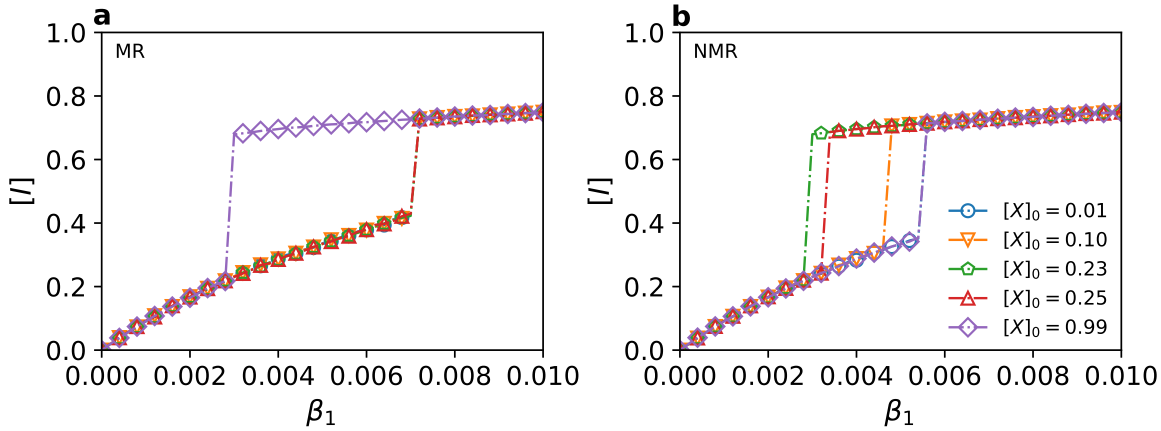

Supplementary Figure 1. **Effects of initial conditions on phase transition**. The initial conditions are $[X]_0 \neq 0$ and $[Y]_0 = 0$. The phase transition is with respect to a systematic increase in the value of parameter β_1. The results are obtained from the same initial conditions for different values of β_1 (for a fixed β_2 value). There is a critical value $\beta_c([X]_0)$ beyond which the system approaches a high-failure state. (a,b) Results for MR and NMR models, respectively, where the blue circles, orange down triangles, green pentagons, red up triangles, purple diamonds represent the results from different initial fractions of failed nodes: $[X]_0 = 0.01, 0.1, 0.23, 0.25, 0.99$, respectively. Other parameters are $\beta_2 = 2$, $\mu_1 = 0.01$, $\mu_2 = 1$, $\tau_1 = 100$, $\tau_2 = 1$, and $m = 15$. The network has a random regular structure with size $N = 3 \times 10^4$ and nodal degree $k = 35$.

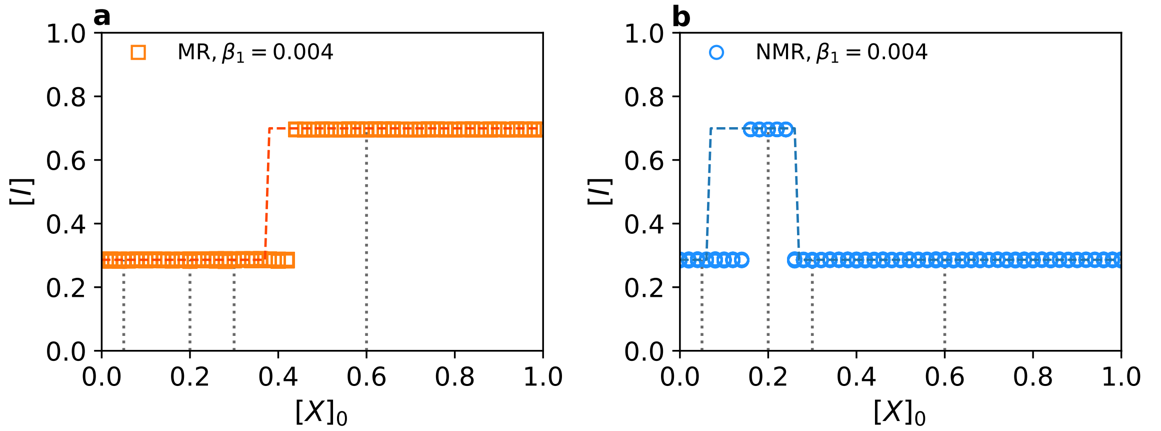

Supplementary Figure 2. **Effects of initial conditions on stationary solution**. The initial conditions are $[X]_0 \neq 0$ and $[Y]_0 = 0$. The stationary solution $[I]$ is obtained for $\beta_1 = 0.004$ for MR (a) and NMR (b) models. Orange squares and blue circles are simulation results for MR and NMR models, respectively. The dashed line represents the mean-field prediction. The gray dotted vertical lines correspond to $[X]_0 = 0.05, 0.2, 0.3, 0.6$, respectively.

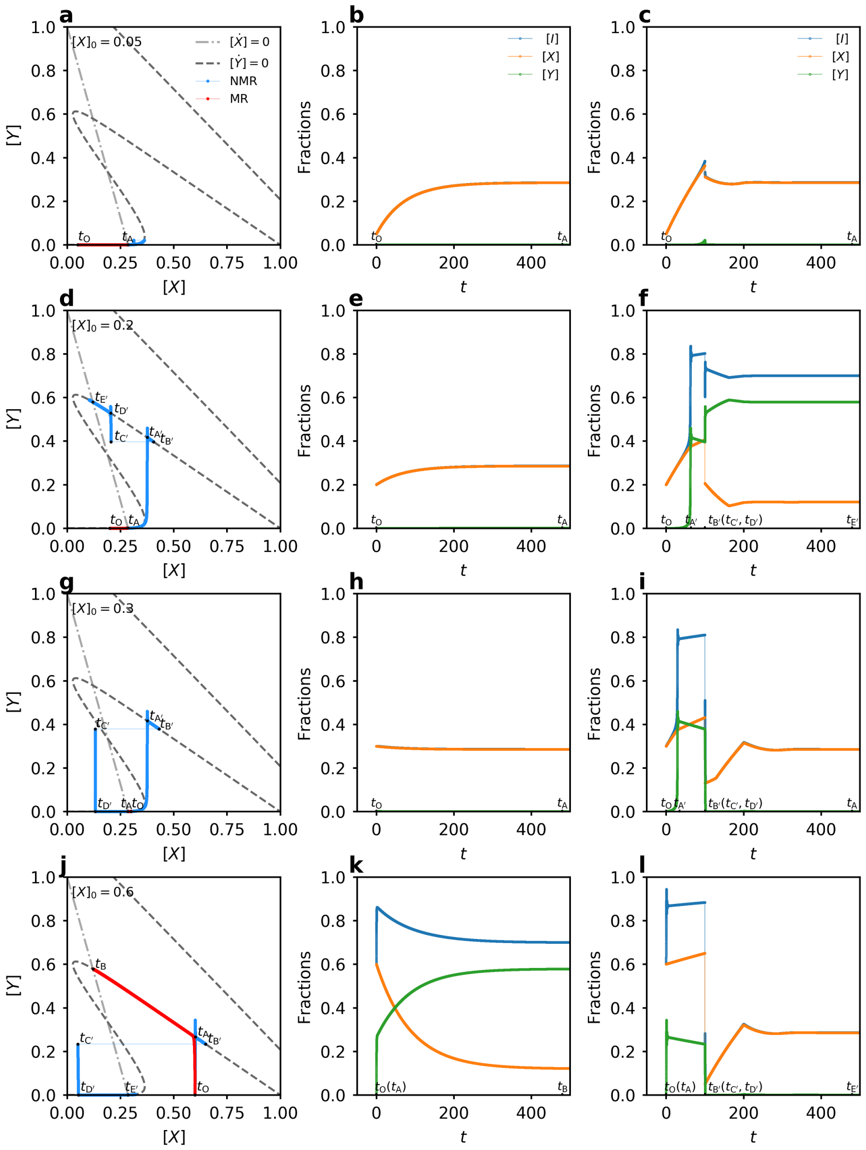

Supplementary Figure 3. **Trajectories and time evolution of fractions of X-type and Y-type nodes**. The initial conditions are $[X]_0 \neq 0$ and $[Y]_0 = 0$. The results are obtained from the mean-field theory. For fixed $[X]_0 = 0.05$, (a) trajectories of $[X]$ and $[Y]$, (b,c) time evolution from the MR and NMR models, respectively. (d-f) The corresponding results for $[X]_0 = 0.2$. (g-i) The results for $[X]_0 = 0.3$. (j-l) The results for $[X]_0 = 0.6$. The solid blue and red lines in the first column are the results from the NMR and MR models, respectively. The light and dark gray dotted lines are the solutions of $[\dot{X}] = 0$ and $[\dot{Y}] = 0$ from the mean-field theory for the MR model, respectively, where their intersections give the steady-state solutions. The solid blue, orange and green lines in the second and third columns are the results of $[I]$, $[X]$ and $[Y]$ for the MR and NMR models, respectively. The evolution from $t_{\mathrm{B}'}$ to $t_{\mathrm{C}'}$ and then to $t_{\mathrm{D}'}$ is too fast to be distinguished in (f), (i) and (l). The evolution from t_{O} to t_{A} is also too short for it to be seen in (k) and (l). Other parameter values are $\beta_1 = 0.004$, $\beta_2 = 2$, $\mu_1 = 0.01$, $\mu_2 = 1$, $\tau_1 = 100$, $\tau_2 = 1$, and $m = 15$. The network is the same as that in Supplementary Fig. 1.

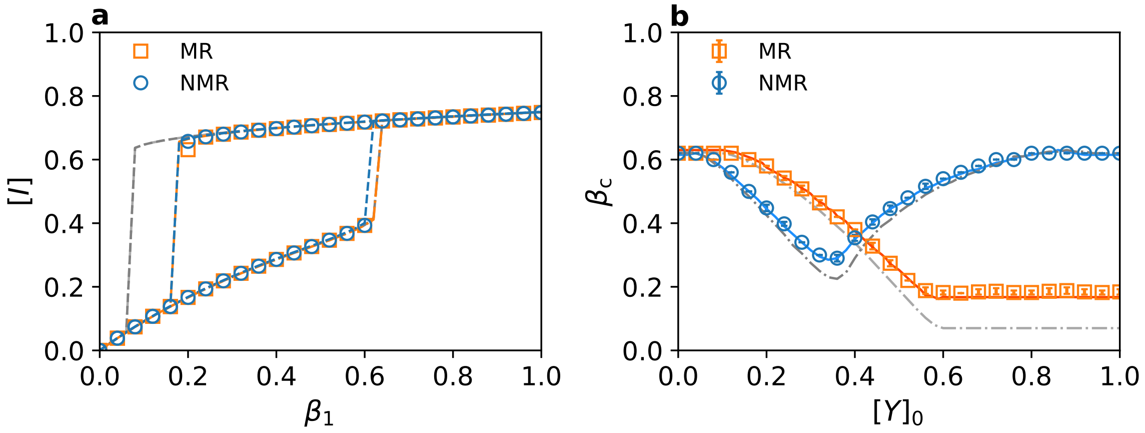

Supplementary Figure 4. **MR and NMR dynamics in situations where external recovery is slower than internal recovery**. (a) Dependence of $[I]$ on β_1 in the steady state for $\beta_2 = 0.1$, $\tau_1 = 1.0$ (corresponding to $\mu_1 = 1.0$), $\tau_2 = 20$ (corresponding to $\mu_2 = 0.05$), and $m = 15$. Orange squares (blue circles) are simulation results for the MR (NMR) model. The results are averaged for two network configurations, each of ten realizations. The orange dot-dashed (blue dashed) line is calculated by the PA theory for the MR (NMR) model. The gray dot-dashed (dashed) line is the result from the MF theory for the MR (NMR) model. (b) Dependence of β_c on the initial value of $[Y]_0$, with $[X]_0 = 0$. The solid (dot-dashed) lines are obtained from the PA (MF) theory. The networks are RRNs with $N = 30000$ and $k = 35$.

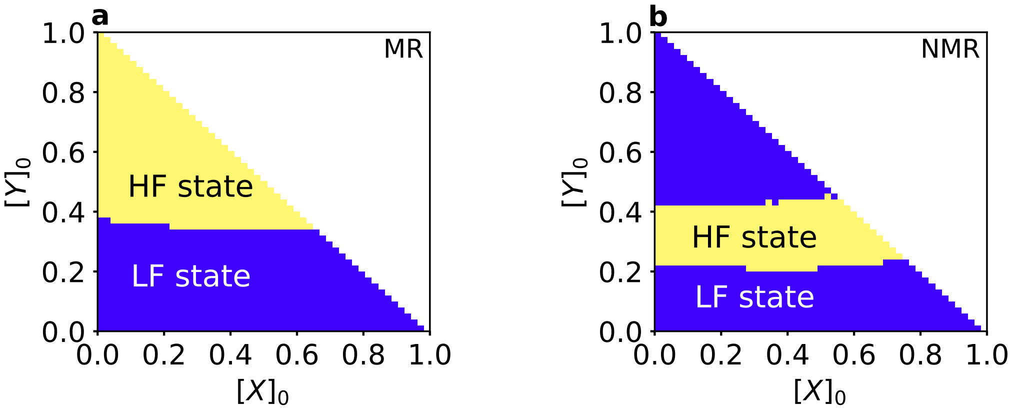

Supplementary Figure 5. **Basin structures**. On the $[X]_0$-$[Y]_0$ plane, basin structure for (a) MR and (b) NMR model for $\beta_1 = 0.4$, where the colors indicate the nature of the steady states from different initial conditions. Other parameters are the same as those in Supplementary Fig. 4.

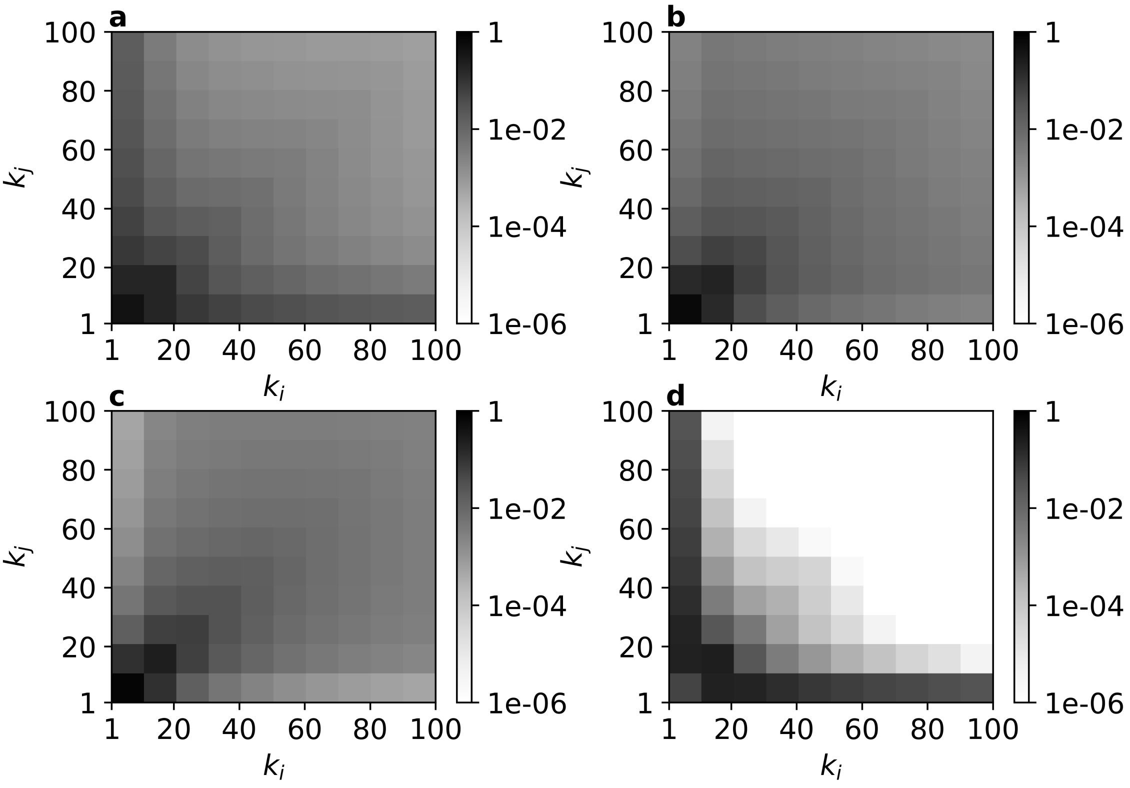

Supplementary Figure 6. **Visualizations of degree-degree correlation in a network**. Shading colors of the matrix correspond to the probabilities that a randomly chosen edge connects nodes i of degree k_i and j of k_j, where the results are averaged with 100 UCN realizations for (a) $r = 0$, (b) $r = 0.5$, (c) $r = 0.7$ and (d) $r = -0.5$. Each grid cell represents the average result with the degree range of ten for visualization. Other network parameters are $N = 10000$, $\gamma = 2.5$, $k_{\min} = 5$, and $k_{\max} = 100$.

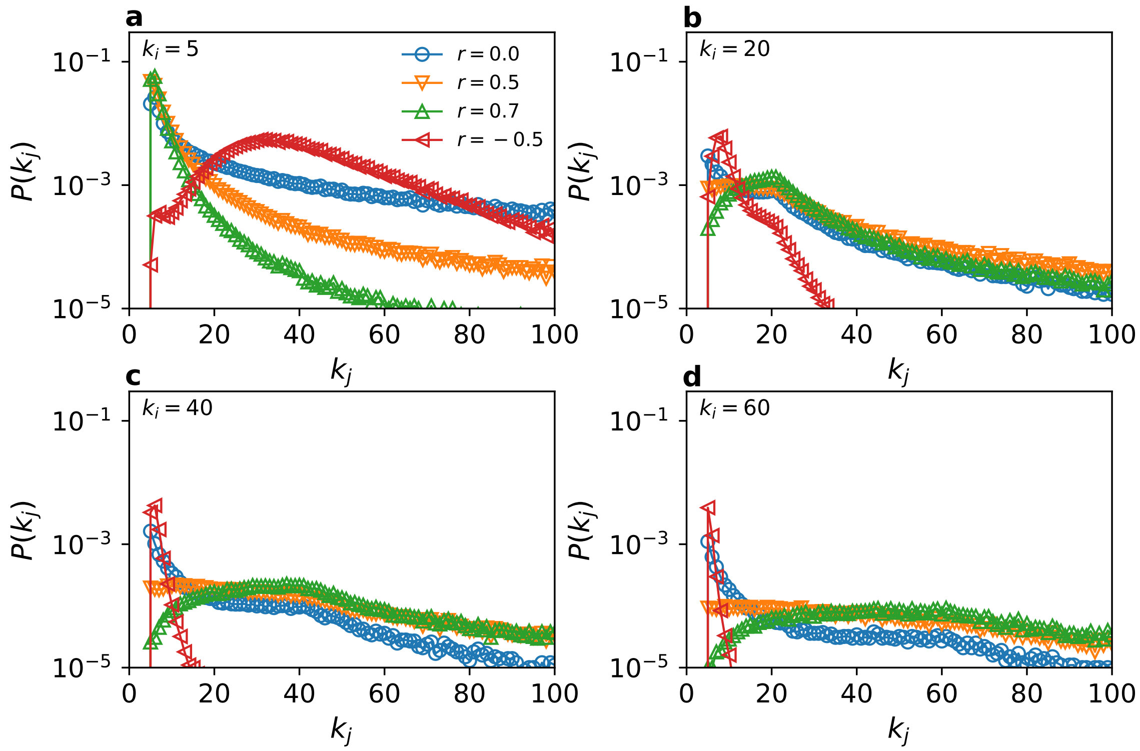

Supplementary Figure 7. **Probability distribution of a node of degree k_i connecting to a node of degree k_j.** (a-d): node i of degree $k_i = 5$, $k_i = 20$, $k_i = 40$ and $k_i = 60$, respectively. Blue circles, orange down triangles, green up triangles and red left triangles are the results for different level of degree-degree correlation $r = 0$, $r = 0.5$, $r = 0.7$ and $r = -0.5$ in Supplementary Figs. 6, respectively.

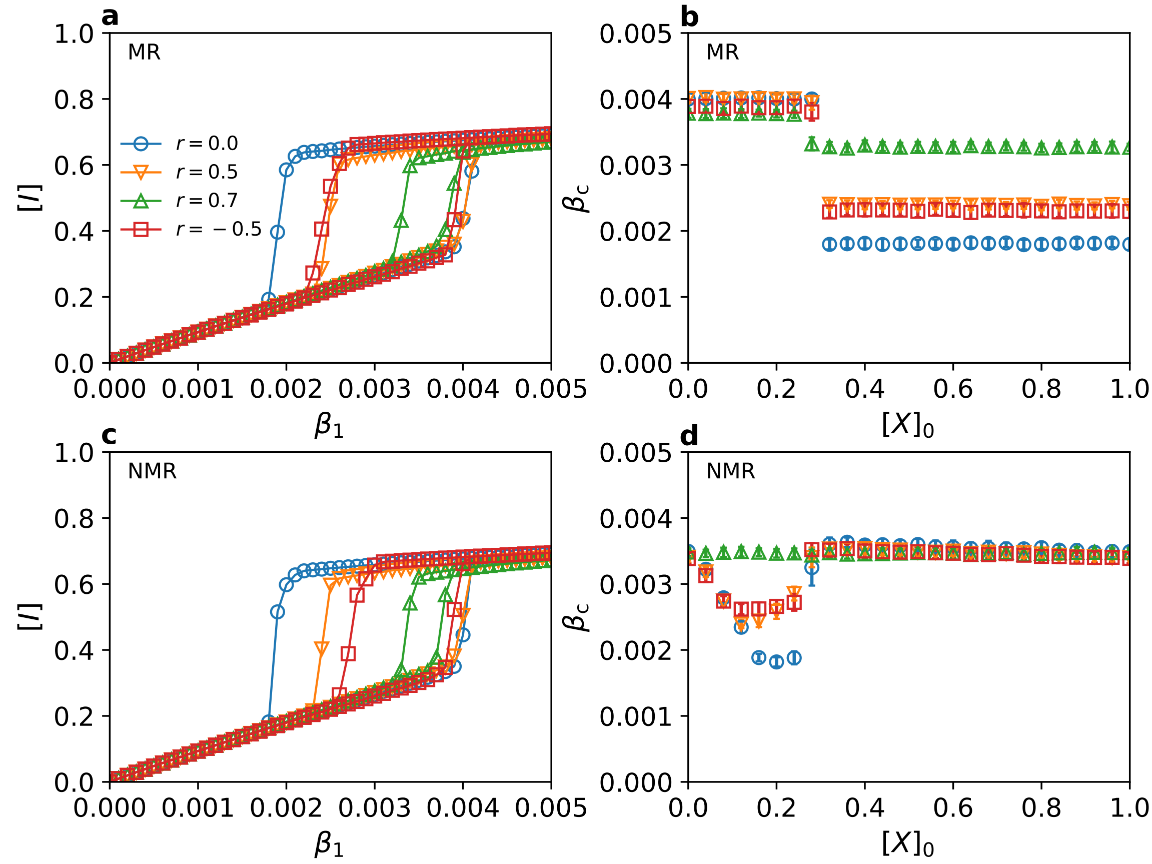

Supplementary Figure 8. **MR and NMR dynamics in UCNs with degree-degree correlation**. (a) Dependence of $[I]$ on β_1 in the steady state for the MR model. The parameter values are $\beta_2 = 2.0$, $\tau_1 = 100$ (corresponding to $\mu_1 = 0.01$), and $\tau_2 = 1.0$ (corresponding to $\mu_2 = 1.0$). The threshold of external failure of a node is that half of its neighbors have failed. Blue circles, orange down triangles, green up triangles and red squares are the simulation results for different values of the degree-degree correlation coefficient: $r = 0$, $r = 0.5$, $r = 0.7$ and $r = -0.5$, respectively. (b) Dependence of β_c on the initial value of $[X]_0$ for the MR model for $[Y]_0 = 0$. (c,d) Simulation results averaged over 50 realizations for the NMR model. The networks are of the UCM type with $N = 10000$, $k_{\min} = 5$, $k_{\max} = 100$, and different levels of degree-degree correlation.

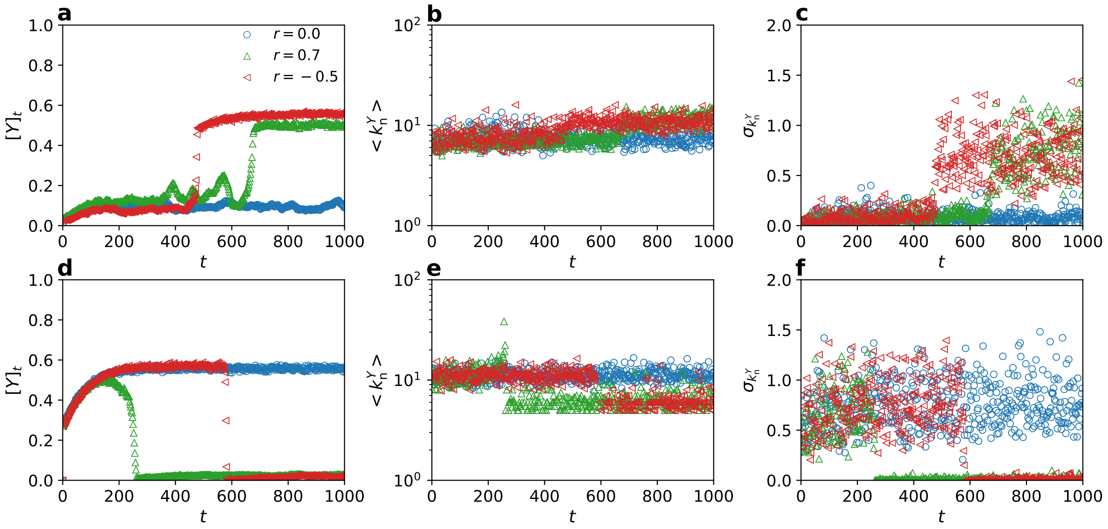

Supplementary Figure 9. **Dynamical behaviors on UCNs with different levels of degree-degree correlation for the MR model**. (a,d) Time evolution of the fraction of Y-type nodes. Blue circles, green up triangles and red left triangles are for $r = 0$, $r = 0.7$ and $r = -0.5$, respectively. (b,e) Time evolution of the average degree of newly emerged Y-type nodes. (c,f) Variance of the newly emerged Y-type nodes whose degrees correspond to those in Supplementary Fig. 9(b). (a-c): Results for $\beta_1 = 0.004$ and $[X]_0 = 0.2$. (d-f) Results for $\beta_1 = 0.0022$ and $[X]_0 = 0.6$. A single realization is used here for better visualization. Other parameter values are the same as those in Supplementary Fig. 8.

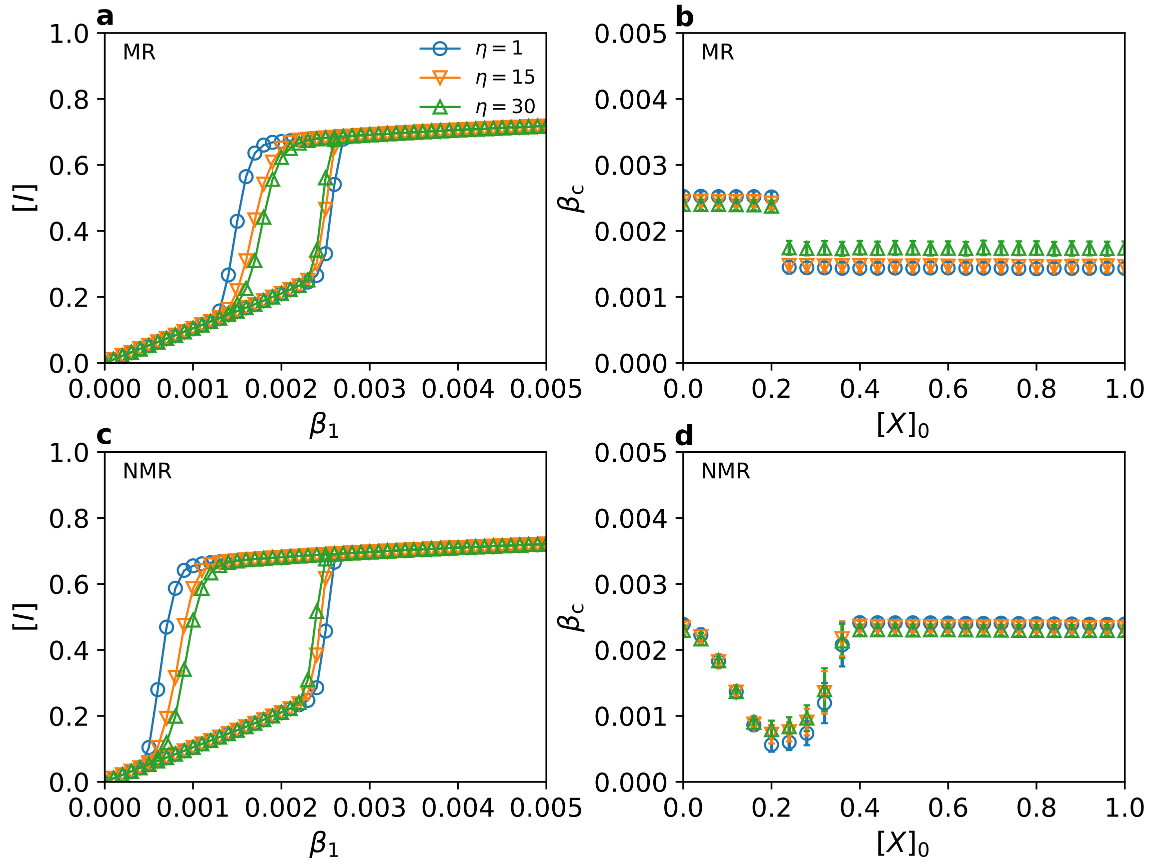

Supplementary Figure 10. **MR and NMR dynamics in networks with a community structure**. (a) Dependence of $[I]$ on β_1 in the steady state for the MR model for $\beta_2 = 2.4$. Blue circles, orange down triangles and green up triangles are simulation results for $Q \approx -0.01$, $Q = 0.43$ and $Q = 0.46$, respectively. Each data point is the result of averaging over 20 network realizations, each with 50 random initial conditions. (b) Dependence of β_c on the initial value of $[X]_0$ for the MR model for $[Y]_0 = 0$. (c,d) The corresponding simulation results for the NMR model. The network size is $N = 3000$ and the mean degree is $\langle k \rangle = 6$. Other parameters are the same as those in Supplementary Fig. 8.

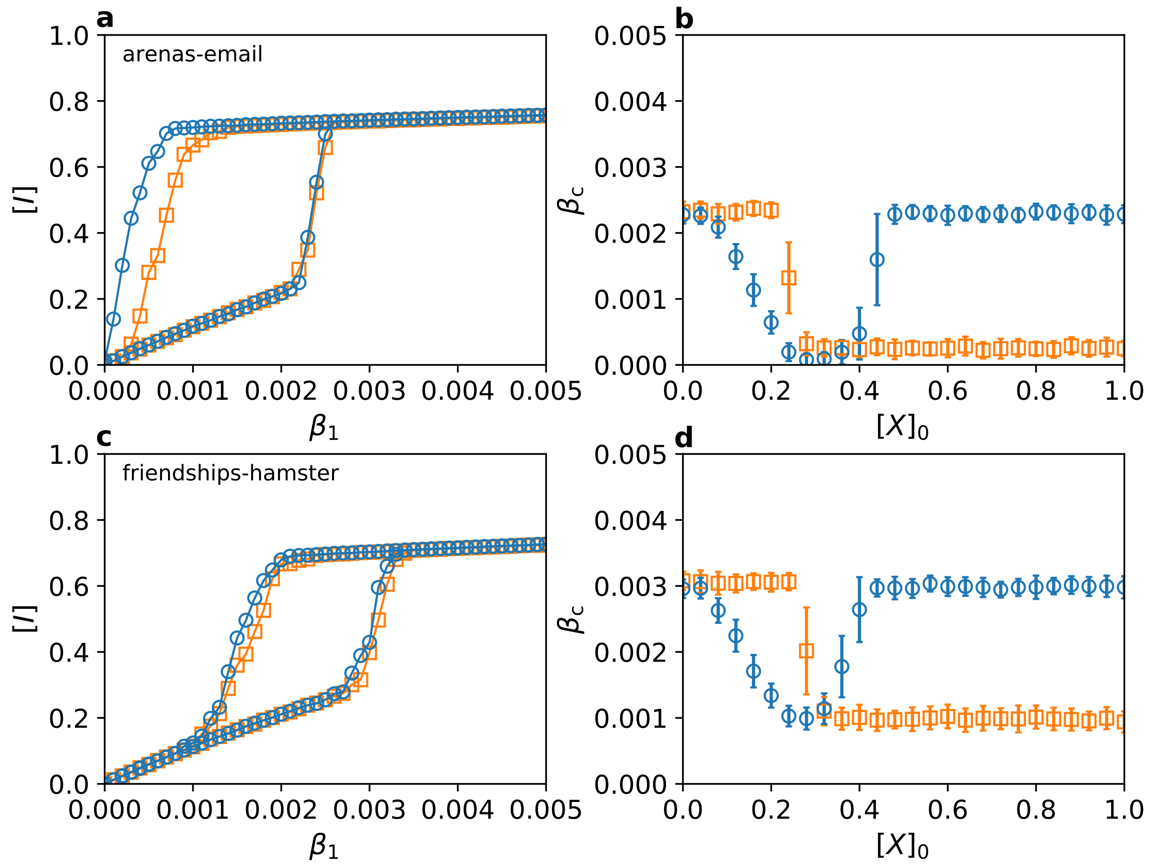

Supplementary Figure 11. **Comparison between MR and NMR dynamics in the empirical networks**. (a) Dependence of $[I]$ on β_1 in the steady state in the arenas-email network for $\beta_2 = 2.9$. Blue circles and orange squares are simulation results averaged over 50 realizations for the MR and NMR dynamical models, respectively. (b) Dependence of β_c on the initial value of $[X]_0$ for the MR and NMR models for $[Y]_0 = 0$. (c,d) The corresponding results in the friendship-hamster network for $\beta_2 = 2.5$. Other parameters are the same as those in Supplementary Fig. 8.

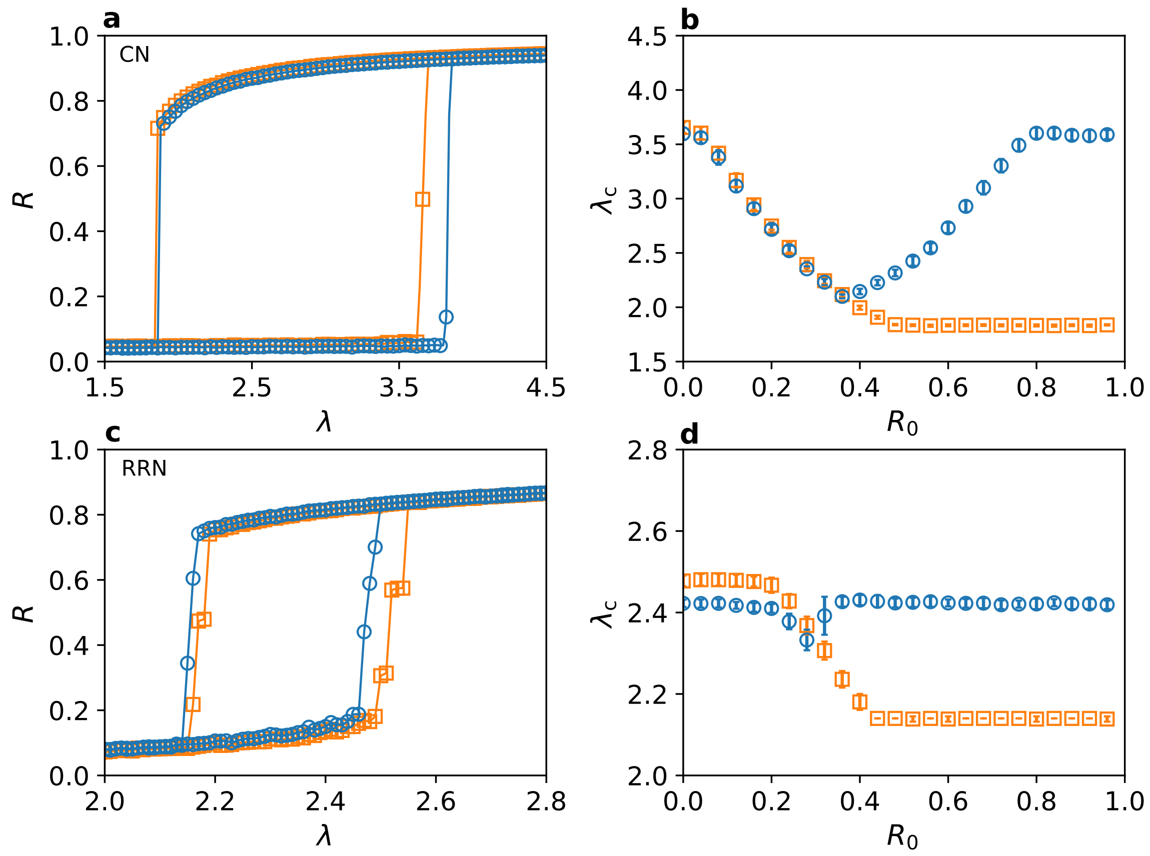

Supplementary Figure 12. **MR and NMR dynamics for power-grid synchronization**. (a,c) Dependence of order parameter R on the coupling strength λ in the steady state for a completely connected network (CN) and a random regular network (RRN), respectively. The results are averaged over 20 realizations. (b,d) Dependence of λ_c on the initial order parameter R_0 for the CN and RRN, respectively. Blue circles and orange squares are for the NMR and MR model, respectively. Other parameters are $a = 0.05$, $p = 0.01$, $\omega_0 = 0$, $\Delta = 0.5$, $\phi = 0$, $\psi = \pi/4$, $N = 5000$, and $\langle k \rangle = 10$ (for RRN).

II. SUPPLEMENTARY NOTES

A. Supplementary Note 1: Pairwise approximation theory

1. Markovian recovery model

We use symbols of the forms $[U]_t$ and $[UV]_t$ with $U, V \in \{A, X, Y\}$ to denote the fractions of nodes and edges in different states at time t, respectively. For example, $[A]_t$, $[X]_t$ and $[Y]_t$ represent the fractions of active nodes, X-type and Y-type failed nodes at time t, respectively, whereas $[AX]_t$ stands for the fraction of active nodes connected with an X-type failed node (i.e., the fraction of AX-type links) at time t. Taking into account every link from every node, the link fractions satisfy the "conservation law": $\sum_{U,V\in\{A,X,Y\}}[UV]_t = 1$, with $[UV]_t = [VU]_t$ for $U \neq V$. The supplementary evolution equations for the fractions of

various types of nodes and edges are given by

$$\frac{d[A]_t}{dt} = \mu_1[X]_t + \mu_2[Y]_t - (\beta_1 + \beta_2 E_t)[A]_t, \tag{1}$$

$$\frac{d[X]_t}{dt} = \beta_1[A]_t - \mu_1[X]_t, \tag{2}$$

$$\frac{d[Y]_t}{dt} = \beta_2 E_t[A]_t - \mu_2[Y]_t, \tag{3}$$

$$\frac{d[AX]_t}{dt} = \mu_1[XX]_t + \mu_2[YX]_t + \beta_1[AA]_t - \mu_1[AX]_t - (\beta_1 + \beta_2 E^{'}_{x,t})[AX]_t, \tag{4}$$

$$\frac{d[AY]_t}{dt} = \mu_1[XY]_t + \mu_2[YY]_t + \beta_2 E^{''}_t[AA]_t - \mu_2[AY]_t - (\beta_1 + \beta_2 E^{'}_{y,t})[AY]_t, \tag{5}$$

$$\frac{d[AA]_t}{dt} = \mu_1([XA]_t + [AX]_t) + \mu_2([YA]_t + [AY]_t) - 2(\beta_1 + \beta_2 E^{''}_t)[AA]_t, \tag{6}$$

$$\frac{d[YY]_t}{dt} = \beta_2 E^{'}_{y,t}([AY]_t + [YA]_t) - 2\mu_2[YY]_t, \tag{7}$$

$$\frac{d[XX]_t}{dt} = \beta_1([AX]_t + [XA]_t) - 2\mu_1[XX]_t, \tag{8}$$

$$\frac{d[XY]_t}{dt} = \beta_1[AY]_t + \beta_2 E^{'}_{x,t}[XA]_t - (\mu_1 + \mu_2)[XY]_t, \tag{9}$$

where

$$E_t = \sum_{j=0}^{m} C_k^{k-j} \left(\frac{[AI]_t}{[A]_t}\right)^{k-j} \left(1 - \frac{[AI]_t}{[A]_t}\right)^j, \tag{10}$$

$$E^{'}_{x,t} = \sum_{j=0}^{m} C_{k-1}^{k-1-j} \left(\frac{[IAX]_t}{[AX]_t}\right)^{k-1-j} \left(1 - \frac{[IAX]_t}{[AX]_t}\right)^j, \tag{11}$$

$$E^{'}_{y,t} = \sum_{j=0}^{m} C_{k-1}^{k-1-j} \left(\frac{[IAY]_t}{[AY]_t}\right)^{k-1-j} \left(1 - \frac{[IAY]_t}{[AY]_t}\right)^j, \tag{12}$$

$$E^{''}_t = \sum_{j=0}^{m-1} C_{k-1}^{k-1-j} \left(\frac{[IAA]_t}{[AA]_t}\right)^{k-1-j} \left(1 - \frac{[IAA]_t}{[AA]_t}\right)^j. \tag{13}$$

In the supplementary evolution equation of $[X]_t$ ($[Y]_t$), the first term represents the fraction of failed A-type nodes due to an internal (external) mechanism, which increases the fraction of the X-type (Y-type) nodes. The second term describes the transition that X-type (Y-type) nodes recover, which decreases the fraction of the X-type (Y-type) nodes.

In the supplementary equation of $[AX]_t$, the first (second) term represents the transition that an X-type (Y-type) node connected with an X-type failed node recovers spontaneously (i.e., become again an A-type node), which increases the fraction of AX edges. The third term represents the situation that the A-type neighbors of the A-type nodes fail due to an internal mechanism, which increases the fraction of AX edges. The forth term describes the transition that X-type nodes at the ends of AX edges recover spontaneously, which decreases

the fraction of AX edges. The last term represents the transition that A-type nodes at the ends of AX edges fail due to an internal or an external mechanism, which decreases the fraction of AX edges. In the supplementary equation of $[AA]_t$, the first (second) term represents the transition that X-type (Y-type) nodes at the ends of AX and XA (AY and YA) edges recover spontaneously, which increases the fraction of AA edges. The third term represents the transition that A-type nodes at the ends of AA edges fail due to the an internal or an external cause (i.e., become X-type or Y-type nodes), which decreases the fraction of AA edges.

From the set of supplementary equations, we see that, under different conditions, the probabilities of an active node satisfying the threshold condition are different. We use the notations E_t, $E^{'}_{x,t}$, $E^{'}_{y,t}$ and $E^{''}_t$ for various probabilities: E_t is the probability for an active node to satisfy the threshold condition, $E^{'}_{x,t}$ ($E^{'}_{y,t}$) is the probability for an A-type node associated with an AX (AY) edge to satisfy the threshold condition, and $E^{''}_t$ denotes the probability that an A-type node connected with an A-type node satisfies the threshold condition. For all the probabilities, the threshold condition is $n_A \leq m$, where n_A is the number of active neighbors.

Using the pairwise approximation

$$[UVW]_t = \frac{[UV]_t[VW]_t}{[V]_t}, \tag{14}$$

we have

$$\frac{[IAX]_t}{[AX]_t} = \frac{[AI]_t}{[A]_t}, \tag{15}$$

$$\frac{[IAY]_t}{[AY]_t} = \frac{[AI]_t}{[A]_t}, \tag{16}$$

and

$$\frac{[IAA]_t}{[AA]_t} = \frac{[AI]_t}{[A]_t}, \tag{17}$$

i.e., $E^{'}_{x,t} = E^{'}_{y,t}$. Letting $E^{'}_{x,t} = E^{'}_{y,t} = E^{'}_t$, we have

$$E_t = \sum_{j=0}^{m} C_k^{k-j} \left(\frac{[AI]_t}{[A]_t}\right)^{k-j} \left(1 - \frac{[AI]_t}{[A]_t}\right)^{j}, \tag{18}$$

$$E^{'}_t = \sum_{j=0}^{m} C_{k-1}^{k-1-j} \left(\frac{[AI]_t}{[A]_t}\right)^{k-1-j} \left(1 - \frac{[AI]_t}{[A]_t}\right)^{j}, \tag{19}$$

and

$$E^{''}_t = \sum_{j=0}^{m-1} C_{k-1}^{k-1-j} \left(\frac{[AI]_t}{[A]_t}\right)^{k-1-j} \left(1 - \frac{[AI]_t}{[A]_t}\right)^{j}. \tag{20}$$

Altogether, in the MR model, Supplementary Eqs. (1-9) describe the failure propagation dynamics.

2. Non-Markovian recovery model

To capture the memory effect of the NMR process, we write the model in terms of supplementary difference equations by decomposing the NMR process into a series of MR processes. In the following, each supplementary difference equations describes the relationship among the fractions of nodes or edges in different states at time $t + \Delta t$ and time t. We invoke the notations $[U]_t$ and $[UV]_t$ with $U, V \in \{A, X, Y\}$ to represent the fractions of nodes and edges of different types at time t, respectively. In addition, we use the notations $[U^l]_t$, $[U^{l_1}V^{l_2}]_t$ and $[U^l V]_t$, where l, l_1 and l_2 represent the passing time of the corresponding nodes being in the current state at time t. Due to symmetry, we have $[AX]_t = [XA]_t$. The supplementary evolutionary equations of the NMR model are given by

$$[A]_{t+\Delta t} = [X^{\tau_1}]_t + [Y^{\tau_2}]_t + (1 - \beta_1 \Delta t - \beta_2 \Delta t E_t)[A]_t, \tag{21}$$

$$[X^l]_{t+\Delta t} = \begin{cases} \beta_1 \Delta t [A]_t, & l \in [0, \Delta t); \\ [X^{l-\Delta t}]_t, & l \in [\Delta t, \tau_1]; \\ 0, & l \in (\tau_1, \infty), \end{cases} \tag{22}$$

$$[Y^l]_{t+\Delta t} = \begin{cases} \beta_2 \Delta t E_t [A]_t, & l \in [0, \Delta t); \\ [Y^{l-\Delta t}]_t, & l \in [\Delta t, \tau_2]; \\ 0, & l \in (\tau_2, \infty), \end{cases} \tag{23}$$

$$[AX^l]_{t+\Delta t} = \begin{cases} \beta_1 \Delta t [AA]_t + \beta_1 \Delta t ([X^{\tau_1} A]_t + [Y^{\tau_2} A]_t), & l \in [0, \Delta t); \\ [X^{\tau_1} X^{l-\Delta t}]_t + [Y^{\tau_2} X^{l-\Delta t}]_t + (1 - \beta_1 \Delta t - \beta_2 \Delta t E'_{x,t})[AX^{l-\Delta t}]_t, & l \in [\Delta t, \tau_1]; \\ 0, & l \in (\tau_1, \infty), \end{cases} \tag{24}$$

$$[AY^l]_{t+\Delta t} = \begin{cases} \beta_2 \Delta t E''_t [AA]_t + \beta_2 \Delta t E'_{y,t} [Y^{\tau_2} A]_t + \beta_2 \Delta t E'_{x,t} [X^{\tau_1} A]_t, & l \in [0, \Delta t); \\ [X^{\tau_1} Y^{l-\Delta t}]_t + [Y^{\tau_2} Y^{l-\Delta t}]_t + (1 - \beta_1 \Delta t - \beta_2 \Delta t E'_{y,t})[AY^{l-\Delta t}]_t, & l \in [\Delta t, \tau_2]; \\ 0, & l \in (\tau_2, \infty), \end{cases} \tag{25}$$

$$\begin{aligned} [AA]_{t+\Delta t} =& (1 - \beta_1 \Delta t - \beta_2 \Delta t E'_t)([X^{\tau_1} A]_t + [AX^{\tau_1}]_t + [Y^{\tau_2} A]_t + [AY^{\tau_2}]_t) \\ &+ ([X^{\tau_1} X^{\tau_1}]_t + [Y^{\tau_2} Y^{\tau_2}]_t + [X^{\tau_1} Y^{\tau_2}]_t + [Y^{\tau_2} X^{\tau_1}]_t) \\ &+ (1 - 2\beta_1 \Delta t - 2\beta_2 \Delta t E''_t)[AA]_t, \end{aligned} \tag{26}$$

$$[Y^{l_1} Y^{l_2}]_{t+\Delta t} = \begin{cases} 0, & l_1 \in [0, \Delta t) \text{ and } l_2 \in [0, \Delta t); \\ \beta_2 \Delta t E'_{y,t} [AY^{l_2 - \Delta t}]_t, & l_1 \in [0, \Delta t) \text{ and } l_2 \in [\Delta t, \tau_2]; \\ \beta_2 \Delta t E'_{y,t} [Y^{l_1 - \Delta t} A]_t, & l_2 \in [0, \Delta t) \text{ and } l_1 \in [\Delta t, \tau_2]; \\ [Y^{l_1 - \Delta t} Y^{l_2 - \Delta t}]_t, & l_1 \text{ and } l_2 \in [\Delta t, \tau_2]; \\ 0, & l_1 \text{ or } l_2 \in (\tau_2, \infty), \end{cases} \tag{27}$$

$$[X^{l_1}X^{l_2}]_{t+\Delta t} = \begin{cases} 0, & l_1 \in [0,\Delta t) \text{ } and \text{ } l_2 \in [0,\Delta t); \\ \beta_1 \Delta t [AX^{l_2-\Delta t}]_t, & l_1 \in [0,\Delta t) \text{ } and \text{ } l_2 \in [\Delta t, \tau_1]; \\ \beta_1 \Delta t [X^{l_1-\Delta t}A]_t, & l_2 \in [0,\Delta t) \text{ } and \text{ } l_1 \in [\Delta t, \tau_1]; \\ [X^{l_1-\Delta t}X^{l_2-\Delta t}]_t, & l_1 \text{ } and \text{ } l_2 \in [\Delta t, \tau_1]; \\ 0, & l_1 \text{ } or \text{ } l_2 \in (\tau_1, \infty), \end{cases} \tag{28}$$

$$[X^{l_1}Y^{l_2}]_{t+\Delta t} = \begin{cases} 0, & l_1 \in [0,\Delta t) \text{ } and \text{ } l_2 \in [0,\Delta t); \\ \beta_1 \Delta t [AY^{l_2-\Delta t}]_t, & l_1 \in [0,\Delta t) \text{ } and \text{ } l_2 \in [\Delta t, \tau_2]; \\ \beta_2 \Delta t E'_{x,t} [X^{l_1-\Delta t}A]_t, & l_2 \in [0,\Delta t) \text{ } and \text{ } l_1 \in [\Delta t, \tau_1]; \\ [X^{l_1-\Delta t}Y^{l_2-\Delta t}]_t, & l_1 \in [\Delta t, \tau_1] \text{ } and \text{ } l_2 \in [\Delta t, \tau_2]; \\ 0, & l_1 \in (\tau_1, \infty) \text{ } or \text{ } l_2 \in (\tau_2, \infty), \end{cases} \tag{29}$$

where

$$E_t = \sum_{j=0}^{m} C_k^{k-j} \left(\frac{[AI]_t}{[A]_t}\right)^{k-j} \left(1 - \frac{[AI]_t}{[A]_t}\right)^j, \tag{30}$$

$$E'_{x,t} = \sum_{j=0}^{m} C_{k-1}^{k-1-j} \left(\frac{[IAX]_t}{[AX]_t}\right)^{k-1-j} \left(1 - \frac{[IAX]_t}{[AX]_t}\right)^j, \tag{31}$$

$$E'_{y,t} = \sum_{j=0}^{m} C_{k-1}^{k-1-j} \left(\frac{[IAY]_t}{[AY]_t}\right)^{k-1-j} \left(1 - \frac{[IAY]_t}{[AY]_t}\right)^j, \tag{32}$$

and

$$E''_t = \sum_{j=0}^{m-1} C_{k-1}^{k-1-j} \left(\frac{[IAA]_t}{[AA]_t}\right)^{k-1-j} \left(1 - \frac{[IAA]_t}{[AA]_t}\right)^j. \tag{33}$$

In addition, we have

$$[AX^l]_t = [X^lA]_t, \tag{34}$$

$$[AY^l]_t = [Y^lA]_t, \tag{35}$$

$$[X^{l_1}Y^{l_2}]_t = [Y^{l_2}X^{l_1}]_t, \tag{36}$$

$$[AI]_t = \sum_{l=0}^{\tau_1} [AX^l]_t + \sum_{l=0}^{\tau_2} [AY^l]_t, \tag{37}$$

$$[AI^l]_t = [AX^l]_t + [AY^l]_t. \tag{38}$$

In the supplementary evolutionary equations of $[X^l]_{t+\Delta t}$, the fraction of X-type nodes with $l \in [0, \Delta t)$ at time $t + \Delta t$ is equal to the fraction of A-type nodes that fail due to internal causes in the time interval $[t, t+\Delta t)$. The fraction of X-type nodes with $\Delta t \le l \le \tau_1$ is equal to the fraction of X-type nodes with $\Delta t \le l - \Delta t \le \tau_1$ at time t. Since l cannot exceed the recovery time, the fraction of X-type nodes with $l > \tau_1$ is zero. The supplementary equations for $[Y^l]_{t+\Delta t}$ can be obtained in a similar way.

For the supplementary equations of $[AX^l]_{t+\Delta t}$, for $l \in [0, \Delta t)$, the first term means that the A-type neighbors of A-type nodes fail due to internal causes, which increases the fraction of AX^l edges with $l \in [0, \Delta t)$ at time $t + \Delta t$. The second (third) term depicts that the A-type nodes connected with an X-type (Y-type) neighbor fail due to internal causes while

their X-type (Y-type) neighbors recover spontaneously (i.e., recover because the recovery time has been reached), which increases the fraction of AX^l edges with $l \in [0, \Delta t)$. For $\Delta t \leq l \leq \tau_1$, the first (second) term describes that the X-type nodes connected with an X-type (Y-type) neighbor recover spontaneously, which increases the fraction of AX^l edges, with $l \in [\Delta t, \tau_1]$ at time $t + \Delta t$. The third term stipulates that there must be no change in the states of $AX^{l-\Delta t}$ edges, i.e., the A-type nodes associated with $AX^{l-\Delta t}$ edges have not failed during the time interval $[t, t + \Delta t)$. For $l > \tau_1$, we have $[AX^l]_{t+\Delta t} = 0$.

In the supplementary equation of $[AA]_{t+\Delta t}$, the first term denotes that, associated with edges $X^{\tau_1}A$, AX^{τ_1}, $Y^{\tau_2}A$ and AY^{τ_2}, the states of A-type nodes are not changed, but the states of the failed nodes have changed, which increases the fraction of AA edges at time $t+\Delta t$. The second term describes that both nodes at the end of $X^{\tau_1}X^{\tau_1}$, $Y^{\tau_2}Y^{\tau_2}$, $X^{\tau_1}Y^{\tau_2}$, and $Y^{\tau_2}X^{\tau_1}$ edges recover, which increases the fraction of AA edges. The third term stipulates that the states of both nodes at the end of AA edges must not change.

Similar to the MR model, we use E_t, $E_t^{'}$, and $E_t^{''}$ to represent the probabilities that an active node satisfies the threshold condition $n \leq m$ in different cases.

Using the PA $[UVW]_t = [UV]_t[VW]_t/[V]_t$, we have

$$\frac{[IAX]_t}{[AX]_t} = \frac{[AI]_t}{[A]_t}, \tag{39}$$

$$\frac{[IAY]_t}{[AY]_t} = \frac{[AI]_t}{[A]_t} \tag{40}$$

and

$$\frac{[IAA]_t}{[AA]_t} = \frac{[AI]_t}{[A]_t}, \tag{41}$$

i.e., $E_{x,t}^{'} = E_{y,t}^{'}$. Letting $E_{x,t}^{'} = E_{y,t}^{'} = E_t^{'}$, we have

$$E_t = \sum_{j=0}^{m} C_k^{k-j} \left(\frac{[AI]_t}{[A]_t}\right)^{k-j} \left(1 - \frac{[AI]_t}{[A]_t}\right)^j, \tag{42}$$

$$E_t^{'} = \sum_{j=0}^{m} C_{k-1}^{k-1-j} \left(\frac{[AI]_t}{[A]_t}\right)^{k-1-j} \left(1 - \frac{[AI]_t}{[A]_t}\right)^j, \tag{43}$$

and

$$E_t^{''} = \sum_{j=0}^{m-1} C_{k-1}^{k-1-j} \left(\frac{[AI]_t}{[A]_t}\right)^{k-1-j} \left(1 - \frac{[AI]_t}{[A]_t}\right)^j. \tag{44}$$

The number of supplementary equations depends on the time step Δt. If Δt is small compared with other time scales in the dynamics, the number of supplementary equations will be large.

3. Relationship between MR and NMR models

For the MR model, the supplementary mean-field equations can be written concisely as

$$\begin{cases} \dfrac{d[X]_t}{dt} = \beta_1[A]_t - \mu_1[X]_t, \\ \dfrac{d[Y]_t}{dt} = \beta_2 E_t[A]_t - \mu_2[Y]_t, \end{cases} \tag{45}$$

where

$$E_t = \sum_{j=0}^{m} C_k^{k-j}([I]_t)^{k-j}(1-[I]_t)^j. \tag{46}$$

For the NMR model, in a compact form, the supplementary mean-field equations are

$$\begin{cases} [X]_{t+\Delta t} = \beta_1 \Delta t[A]_t + [X]_t - [X^{\tau_1}]_t, \\ [Y]_{t+\Delta t} = \beta_2 \Delta t E_t[A]_t + [Y]_t - [Y^{\tau_2}]_t, \end{cases} \tag{47}$$

where

$$E_t = \sum_{j=0}^{m} C_k^{k-j}([I]_t)^{k-j}(1-[I]_t)^j. \tag{48}$$

When the system has reached a steady state ($t \to \infty$), we have $[\dot{A}]_t = 0$, $[\dot{X}]_t = 0$ and $[\dot{Y}]_t = 0$. Supplementary Eq. (47) can be written as

$$\begin{cases} \beta_1[A]_t - [X^{\tau_1}]_t = 0, \\ \beta_2 E_t[A]_t - [Y^{\tau_2}]_t = 0. \end{cases} \tag{49}$$

From Supplementary Eq. (49), we have

$$[X^{\tau_1}]_t = \beta_1[A]_t = C_1 \tag{50}$$

and

$$[Y^{\tau_2}]_t = \beta_2 E[A]_t = C_2, \tag{51}$$

where C_1 and C_2 are constants. In addition, we have

$$[X^l]_t = [X^{\tau_1}]_{t+\tau_1-l} = C_1 \tag{52}$$

and

$$[Y^l]_t = [Y^{\tau_2}]_{t+\tau_2-l} = C_2. \tag{53}$$

We then have

$$[X]_t = \Sigma_l^{\tau_1}[X^l]_t = \tau_1[X^{\tau_1}]_t, \tag{54}$$

which gives

$$[X^{\tau_1}]_t = \frac{1}{\tau_1}[X]_t. \tag{55}$$

Similarly, we can get

$$[Y^{\tau_2}]_t = \frac{1}{\tau_2}[Y]_t. \tag{56}$$

Supplementary Equation (49) can then be rewritten as

$$\begin{cases} \beta_1[A]_t - \dfrac{1}{\tau_1}[X]_t = 0, \\ \beta_2 E_t[A]_t - \dfrac{1}{\tau_2}[Y]_t = 0. \end{cases} \tag{57}$$

Comparing with the steady-state solution of the MR model:

$$\begin{cases} \beta_1[A]_t - \mu_1[X]_t = 0, \\ \beta_2 E_t[A]_t - \mu_2[Y]_t = 0, \end{cases} \tag{58}$$

we see that the steady states for both models are equivalent to each other for fixed values of $\mu_1 = 1/\tau_1$ and $\mu_2 = 1/\tau_2$.

B. Supplementary Note 2: Effects of different initial conditions on failure propagation and evolutionary trajectories of failed nodes

Supplementary Figs. 1 and 2 illustrate the effects of initial conditions on failure propagation dynamics. The different recovery mechanisms make the competition processes between the X-type and Y-type nodes distinct for the MR and NMR models. In general, non-Markovian features render more complicated the failure-recovery dynamics. Here we investigate how the fraction of initially failed nodes $[I]_0$ influences the phase transition in the NMR model.

We set the initial conditions as $[X]_0 \neq 0$ and $[Y]_0 = 0$. When the fraction of initially failed nodes is sufficiently small, e.g., $[X]_0 = 0.05$, as in Supplementary Fig. 3(a), active nodes fail most likely because of internal causes. In both MR and NMR models, the system will evolve into a low-failure state (phase), where the trajectories evolve from t_{O} to t_{A}. As $[X]_0$ increases to 0.2, as shown in Supplementary Fig. 3(d), the evolutionary process for the MR model is similar to the case in Supplementary Fig. 3(a): the trajectory evolves from t_{O} to t_{A}. This is because the X-type nodes not only are born at the rate β_1 but also recover at the rate μ_1, driving the system to a dynamic equilibrium associated with the low-failure phase. As a result, there are insufficient newly created X-type nodes to cause a large scale external failure. In contrast, in the NMR model, X-type nodes are always created but, in the early stage $[t_{\text{O}}, t_{\text{A}}]$, there is no recovery because of the memory effect. Once the fraction of failed nodes, including the initial and the newly created X-type nodes in $[t_{\text{O}}, t_{\text{A}}]$, reaches a critical value determined by the criterion that many A-type nodes with active neighbors satisfy the threshold condition $n_A \leq m$, $[Y]$ increases rapidly and the trajectory moves from t_{A} to $t_{\text{A}'}$. In the time interval $[t_{\text{A}'}, t_{\text{B}'}]$, $[Y]$ decreases slowly due to a short recovery time $\tau_2 = 1$, while internal failures make $[X]$ increase slowly because $t_{\text{B}'} < 100$. In the time interval $t \in [100, 100 + \Delta t)$, the age of the initially failed nodes ($[I]_0 = [X]_0 = 0.2$) reaches the recovery time and these nodes will turn into A state simultaneously. Consequently, $[X]$ decreases suddenly to a low value and the trajectory evolves sharply from $t_{\text{B}'}$ to $t_{\text{C}'}$. At this time, $[I]$ still remains at a high value, making more active nodes (including the original and new X-type nodes) satisfy the threshold condition $n_A \leq m$ and resulting in a rapid growth of Y-type nodes in the time interval $[t_{\text{C}'}, t_{\text{D}'}]$. At time $t_{\text{D}'}$, $[I]$ increases to a higher

value, rendering more competitive Y than X state as they compete for A-type nodes. As a result, A-type nodes fail due mostly to external causes, and $[Y]$ ($[X]$) continues to increase (decrease) in the time interval $[t_{\mathrm{D}'}, t_{\mathrm{E}'}]$.

For $[X]_0 = 0.3$, as shown in Supplementary Fig. 3(g), the evolutionary process of the MR model is consistent with that in Supplementary Fig. 3(d). For the NMR model, the evolutionary process is different from that in Supplementary Fig. 3(b). In the time interval $[t_{\mathrm{B}'}, t_{\mathrm{C}'}]$, more initially failed nodes with $[I]_0 = [X]_0 = 0.3$ recover simultaneously because their age (i.e., the current time) has reached the recovery time $\tau_1 = 100$. At time $t_{\mathrm{C}'}$, a relatively low value of $[I]$ makes fewer active nodes satisfy the threshold condition $n_A \leq m$. Because of the short recovery time, all the current Y-type nodes switch into the active state in the time interval $[t_{\mathrm{C}'}, t_{\mathrm{D}'}]$. After that, many active nodes fail internally and become X-type nodes, so $[X]$ increases continuously and the trajectory moves from $t_{\mathrm{D}'}$ to t_{A}.

When the initial value $[X]_0$ is sufficiently large, e.g., 0.6 as in Supplementary Fig. 3(j), many A-type nodes will fail due to external causes and $[Y]$ will increase rapidly in both models, as shown in the trajectory from t_{O} to t_{A}. After that, in the MR model, the increment of $[Y]$ enhances the probability for active nodes to switch into the Y state, while increasingly X-type nodes recover and turn to the A state. The corresponding trajectory moves from t_{A} to t_{B}. In the NMR model, the evolutionary process is similar to that in Supplementary Fig. 3(g).

C. Supplementary Note 3: Markovian and non-Markovian dynamics when external recovery is slower than internal recovery

In the main text, the case where internal recovery is slower than external recovery, i.e., $\tau_1 > \tau_2$, is treated. There are situations in the real world where the opposite, i.e., $\tau_1 < \tau_2$, can occur. For example, in the case of an earthquake, the recovery of a node in an infrastructure (e.g., restoring a damaged building) may require more time than that of repair due to internal material failures. To study this case, we fix the network topology as in Supplementary Fig. 1. Supplementary Fig. 4 shows the representative results. Comparing Supplementary Fig. 4(a) with Fig. 2(b) in main text, we see that they demonstrate qualitatively similar behaviors, indicating the applicability of our theoretical framework for the case $\tau_1 < \tau_2$.

Supplementary Fig. 5 shows the phase diagram on the initial-condition plane $([X]_0, [Y]_0)$. Comparing it with Fig. 7 in the main text, we see that both exhibit similar phenomena, although the quantitative details are different. This further validates that our developed framework is applicable to the case $\tau_1 < \tau_2$.

D. Supplementary Note 4: Effects of network structure on Markovian and non-Markovian recovery dynamics

In general, network topology can have a significant effect on the cascading process of failure propagation. In the main text, we have discussed two kinds of network topology, i.e., random regular and scale-free networks, and found that memory in the nodal recovery can counterintuitively make both types of networks more resilient against large scale failures. Here we study two additional types of network: those with degree-degree correlation and a

community structure, respectively, and demonstrate the same effect of memory.

1. Effects of degree-degree correlation

To generate networks with adjustable degree-degree correlation coefficients, we use the standard edge-rewiring procedure [1, 2]. In particular, we first generate an uncorrelated configuration network (UCN) [3] with the degree range $[k_{\rm min}, \sqrt{N}]$ and degree distribution $P(k) \sim k^{-\gamma}$. Keeping the degree of each node unchanged, we adjust the degree-degree correlation through the following process. Firstly, at each step, we randomly choose two edges in the network, disconnect them, and switch the two links among the four chosen nodes. Secondly, to generate an assortative (a disassortative) network, we add a new edge between the highest degree and the second highest (lowest) nodes and then connect the remaining pair of nodes. If either of the new edges already exists, we leave the network unchanged. Thirdly, we repeat the process until the observed degree-degree correlation coefficient has reached a target value, which is defined as [4]:

$$r = \frac{\sum_{ij}(A_{ij} - k_i k_j/2m_{\rm e})k_i k_j}{\sum_{ij}(k_i \delta_{ij} - k_i k_j/2m_{\rm e})k_i k_j}, \tag{59}$$

where $m_{\rm e}$ is the total number of edges in the network, $A_{ij} = 1$ if there is an edge between the node i and j (otherwise, $A_{ij} = 0$), δ_{ij} is the Kronecker delta (1 if $i = j$ and 0 otherwise). There is no degree-degree correlation for $r = 0$, but the network will have positive (negative) degree-degree correlation for $r > 0$ ($r < 0$).

Supplementary Figs. 6(a-d) show the degree-degree correlation properties of the UCNs for $r = 0$, $r = 0.5$, $r = 0.7$ and $r = -0.5$, respectively, where it can be visually seen that the high-degree nodes in the network with a positive correlation tend to be connected together, while a high-degree node in the case of negative correlation tends to be connected to a low-degree one. Supplementary Fig. 7 shows the probability distribution of one node of degree k_i connecting to another node of degree k_j, where panels (a-d) correspond to the cases of $k_i = 5$, $k_i = 20$, $k_i = 40$ and $k_i = 60$, respectively.

Supplementary Fig. 8 shows the results of spontaneous recovery dynamics on UCNs with different levels of degree-degree correlation for $N = 10000$, $k_{\rm min} = 5$, and $k_{\rm max} = 100$. Supplementary Figs. 8(a) and 8(c) show the dependence of $[I]$ on adiabatic variations in β_1 in the steady state for the MR and NMR models, respectively. The blue circles, orange down triangles, green up triangles and red squares are the average simulation results for degree-degree correlation coefficient $r = 0$, $r = 0.5$, $r = 0.7$ and $r = -0.5$, respectively, where the system is regarded as in the low-failure phase when β_1 is smaller than the critical value $\beta_{\rm c}$ and a large scale failure occurs when β_1 exceeds $\beta_{\rm c}$. For the case of zero degree-degree correlation, the network can remain in the low-failure phase for the largest possible value of $\beta_{\rm c}$, indicating the highest possible degree of resilience. However, in the high-failure phase with adiabatically decreasing value of β_1, the network with a high level of degree-degree correlation has a larger value of $\beta_{\rm c}$, signifying a stronger ability to recover from damage. As a result, in spontaneous recovery models, UCNs with degree-degree correlations can make the hysteresis region smaller, regardless of positive or negative degree-degree correlation. Supplementary Figs. 8(b) and 8(d) present further evidence to support the finding in the

main text that non-Markovian recovery makes the networks more resilient against large scale failures, regardless of the detailed network structure.

To explain why positive or negative degree-degree correlation shrinks the hysteresis region in Fig. 8, we focus on the MR model. Supplementary Fig. 9(a) shows the time evolution of the fraction of Y-type nodes for $\beta_1 = 0.004$ and $[X]_0 = 0.2$, where the the system is in the low-failure phase. The degree correlation tends to reduce the resilience of system, thereby promoting cascading failures. Corresponding to the steady-state of $[I]$ in Supplementary Fig. 8(a), we see that the large scale failure results from the sharp increase of $[Y]$. Supplementary Figs. 9(b) and 9(c) show the evolution of the mean degree of the newly emerged Y-type nodes and the standard variance of the nodal degree, respectively. It can be seen that, for the networks with $r = 0.7$ (-0.5), the degree values of many newly emerged Y-type nodes are similar for $t < 650$ ($t < 470$). However, after that, the degrees of newly emerged node vary. While the results of both cases look similar, the underlying topological properties of the network are different. In particular, in scale-free networks with a highly heterogeneous degree distribution, a large proportion of the nodes have smaller degrees than the mean degree, but a few hubs have larger degrees. For an assortative network, nodes of similar degree are more likely to be connected together. As a result, there are more vulnerable nodes of low degrees connecting to each other [e.g., the nodes with $k_i = 5$ as shown in Supplementary Fig. 7(a)], which comprise a sizable vulnerable component, making the network more susceptible to a large scale failure [5]. When the threshold on external failure (i.e., the critical fraction of the active neighbors) is fixed (e.g., 0.5), a low-degree node is vulnerable because it is more easily affected by its neighboring nodes. For a disassortative network, the high-degree nodes prefer to connect to the vulnerable nodes of low degrees [e.g., the nodes of $k_i = 20$ shown in Supplementary Fig. 7(b)], making the hubs more vulnerable against the failures of their neighboring nodes and promoting cascading failures.

Supplementary Fig. 9(d) shows the time evolution of $[Y]_t$ for the MR model for $\beta_1 = 0.0022$ and $[X]_0 = 0.6$, corresponding to the case where the degree correlation suppresses the occurrence of cascading failure when the system is in the high-failure phase. From Supplementary Figs. 9(d-f), we see that for the cases of $r = 0.7$ and $r = -0.5$, there are a large number of externally failed nodes of various degrees in the transient process, after which the system undergoes a sharp decrease in $[Y]_t$ near $t = 250$ and $t = 570$, respectively, leaving few externally failed nodes with a similar degree. The reason that the fraction of $[Y]_t$ decreases sharply is also the vulnerable components. For a small value of β_1, the recovery of a few vulnerable nodes will cause successive recovery of the other connected vulnerable nodes and eventually block failure propagation. Note that, for $r = 0$, the high-failure phase continues, because a more stable high-failure phase depends on the structure in which nodes of quite distinct degrees are connected together, as verified by the results in Supplementary Figs. 6 and 9(f).

2. Effects of community structure

For simplicity, we consider networks that contain two two communities [6, 7], where each community is composed of n nodes and so the network size is $N = 2n$. Nodes in the same community are connected to each other with the probability p_{in}, while links across the two communities occur with the probability p_{out}, so the ratio of the numbers of edges in and

between the communities is $\eta \approx p_{\rm in}/p_{\rm out}$. The degree distributions of the subnetwork in each community and of the whole network are Poisson. Note that the value of η determines the strength of the community structure. In particular, $\eta = 1.0$ means that the numbers of edges in and between communities are the same so, effectively, there is no community structure. Likewise, a value of η much greater than one indicates a stronger community structure with significantly more edges within the individual communities. Quantitatively, the community strength can be measured by the modularity Q defined as [4]

$$Q = \frac{1}{2m_{\rm e}} \sum_{ij} (A_{ij} - \frac{k_i k_j}{2m_{\rm e}}) \delta(c_i, c_j), \tag{60}$$

where $m_{\rm e}$ is the total number of edges in the network, A_{ij} are the elements of the network adjacency matrix, c_i is the community to which node i belongs, and δ_{ij} denotes the Kronecker delta. The value of Q is strictly less than 1.0, where $Q > 0$ ($Q < 0$) means that there are more (less) edges between nodes in the same communities than can be expected by chance.

In our numerical simulations, we fix $N = 3000$ and mean degree $\langle k \rangle = 6$, and generate three typical networks with $\eta = 1.0$, 15.0 and 30.0, with the corresponding approximate Q values -0.01, 0.43 and 0.46, respectively. Initially, we randomly distribute seeds in the whole network. Supplementary Fig. 10 demonstrates the dynamical behaviors for both the MR and NMR models. It can be seen that, if the system is in a low-failure (high-failure) phase, the community structure tends to reduce (enhance) resilience. The reason is that nodes in the same community tend to be connected more closely and thus constitute a high clustering structure. The failure of a node due to an external mechanism tends to enhance the probability for other nodes in the same community to fail. Similarly, if a node has recovered, the probability for other nodes in the same community to recover is increased. As a result, when the system is in the low failure phase, the community structure makes a large scale failure more likely. In contrast, if the system is in the high failure phase, the community structure can facilitate recovery. Qualitatively, these results are consistent with those in Supplementary Note 4.

E. Supplementary Note 5: Markovian and non-Markovian recovery dynamics in empirical networks

We study Markovian and non-Markovian recovery dynamics in empirical networks, using the arenas-email and the friendship-hamster networks as two examples, where the former is an email communication network [8] of size $N = 1133$ and average degree $\langle k \rangle = 9.6$, and the latter is the network of friendship among the users of the website hamsterster.com [9] with $N = 1858$ and $\langle k \rangle = 13.4$. Simulation results reveal the same phenomenon as in other cases, i.e., non-Markovian type of recovery with a memory tends to enhance the network resilience against large scale failures, as shown in Supplementary Fig. 11.

F. Supplementary Note 6: Markovian and non-Markovian recovery dynamics in power-grid synchronization

An idealized model for a power grid is the network of Kuramoto oscillators, where the weighted coupling coefficient between two oscillators is related to their own natural frequencies [10, 11]. We study the following model [12]:

$$\frac{d\theta_i}{dt} = \omega_i + \frac{\lambda|\omega_i|}{k_i}\sum_{j=1}^{N} A_{ij}\sin(\theta_j - \theta_i), \quad i = 1, \ldots, N\ , \tag{61}$$

where ω_i is the natural frequency of oscillator i randomly chosen from the Lorentzian distribution

$$g(\omega) = \frac{1}{\pi}[\frac{\Delta}{(\omega-\omega_0)^2 + \Delta^2}], \tag{62}$$

λ is the overall coupling strength, $k_i = \sum_{j=1}^{N} A_{ij}$ is the degree of node i, and A_{ij} are the elements of the symmetric adjacency matrix. In this model, explosive synchronization [12, 13] can arise. In the regime of complete synchronization, all oscillators are localized in the same area. To investigate the propagation of synchronization under the MR and NMR dynamics, we fix the synchronization area and construct the following dynamical system of phase oscillators:

$$\frac{d\theta_i}{dt} = \omega_i + \frac{\lambda|\omega_i|}{k_i}\sum_{j=1}^{N} A_{ij}\sin(\theta_j - \theta_i) - a\sin(\theta_i - \phi), \quad i = 1, \ldots, N\ , \tag{63}$$

where a is a parameter characterizing the degree of synchronization. The first and third terms on the right-hand side describe the self-dynamics of node i, while the second term represents the interactions between node i and its interacting partners. To see the meaning of ϕ, we introduce the order parameter R

$$Re^{i\Psi} = \frac{1}{N}\sum_{j=1}^{N} e^{i\theta_j}, \tag{64}$$

where $0 \leq R \leq 1$ and Ψ denotes the average phase. The synchronization region can then be defined as $[\phi - \psi, \phi + \psi]$. For the MR dynamics, each oscillator in the synchronized region has the probability p to return to the asynchronous state with a random phase between $[-\pi, \pi]$. For the NMR process, each oscillator can stay in the synchronized region for time $\tau = 1/p$ before leaving the region to become asynchronous with others.

We carry out synchronization simulations on a completely connected network (CN) and a random regular network (RRN) with average degree $\langle k \rangle = 10$. We find that, for both networks, the system transitions to the high-failure (low-failure) phase when increasing (decreasing) λ towards a critical value λ_c. Supplementary Fig. 12 shows the dynamic behaviors for both the MR (orange squares) and NMR (blue circles) dynamics. It can be seen that the results are qualitatively similar to those in either Supplementary Fig. 10 or Supplementary Fig. 11, indicating that non-Markovian recovery makes the network more resilient against large scale breakdown of synchronization. The results also indicate that the network topology can have a considerable impact on the system resilience.

III. SUPPLEMENTARY REFERENCES

[1] Xulvi-Brunet, R. & Sokolov, I. M. Reshuffling scale-free networks: From random to assortative. *Phys. Rev. E* **70**, 066102 (2004).

[2] Gao, L., Wang, W., Pan, L., Tang, M. & Zhang, H.-F. Effective information spreading based on local information in correlated networks. *Sci. Rep.* **6**, 38220 (2016).

[3] Catanzaro, M., Boguná, M. & Pastor-Satorras, R. Generation of uncorrelated random scale-free networks. *Phys. Rev. E* **71**, 027103 (2005).

[4] Newman, M. *Networks* (Oxford University Press, 2010).

[5] Payne, J. L., Dodds, P. S. & Eppstein, M. J. Information cascades on degree-correlated random networks. *Phys. Rev. E* **80**, 026125 (2009).

[6] Girvan, M. & Newman, M. E. Community structure in social and biological networks. *Proc. Natl. Acad. Sci. (USA)* **99**, 7821–7826 (2002).

[7] Condon, A. & Karp, R. M. Algorithms for graph partitioning on the planted partition model. *Random Stru. Algo.* **18**, 116–140 (2001).

[8] U. Rovira i Virgili network dataset – KONECT (2017). URL `http://konect.cc/networks/arenas-email`.

[9] Hamsterster friendships network dataset – KONECT (2017). URL `http://konect.cc/networks/petster-friendships-hamster`.

[10] Dorfler, F. & Bullo, F. Synchronization and transient stability in power networks and nonuniform Kuramoto oscillators. *SIAM J. Cont. Opt.* **50**, 1616–1642 (2012).

[11] Wang, H. & Li, X. Synchronization and chimera states of frequency-weighted Kuramoto-oscillator networks. *Phys. Rev. E* **83**, 066214 (2011).

[12] Zhang, X., Hu, X., Kurths, J. & Liu, Z. Explosive synchronization in a general complex network. *Phys. Rev. E* **88**, 010802 (2013).

[13] Hu, X. *et al.* Exact solution for first-order synchronization transition in a generalized kuramoto model. *Sci. Rep.* **4**, 7262 (2014).
